# The Impact of Perceived Emotional Intelligence on Occupational Stress Among Nurses: Empirical Evidence From a Saudi Health Cluster

**DOI:** 10.1155/2024/8876168

**Published:** 2024-11-16

**Authors:** Abdulaziz M. Alsufyani, Mohammed S. Almalki, Khaled A. Khader, Penelope Stanford, Samantha Freeman, Yasir M. Alsufyani

**Affiliations:** ^1^Comprehensive Rehabilitation Center, Ministry of Human Resources and Social Development, Taif, Saudi Arabia; ^2^College of Nursing, Taif University, Taif, Saudi Arabia; ^3^Division of Nursing, Midwifery and Social Work, School of Health Sciences, Faculty of Biology, Medicine and Health, University of Manchester, Manchester, UK; ^4^College of Nursing, King Khaled University, Abha, Saudi Arabia

**Keywords:** occupational stress, perceived emotional intelligence, stress management, work environment

## Abstract

**Background:** Occupational stress is a significant challenge for healthcare systems worldwide. It compromises the quality of healthcare and jeopardizes patient safety. Globally, the estimated economic impact of occupational stress in the healthcare system ranges from US $221.13 million to US $187 billion. Emotional intelligence has been recognized as a behavioral buffer against occupational stress. Hence, this study investigated whether nurses' perceptions of emotional intelligence impact their self-perceived occupational stress.

**Design:** A predictive correlational design was utilized.

**Method:** A prior power analysis using G ∗ Power 3.1 was conducted. A convenience sample of 734 nurses was recruited from Taif Health Cluster. Data were coded and analyzed using IBM® SPSS® Statistics for Windows v.25. Descriptive and inferential statistics were used. A hierarchical regression technique was used. The level of significance was established at *p* < 0.05. The process of data collection started at August 2022 and continued through October 2022.

**Results:** The results indicated that nurses' age and working area predicted occupational stress perceptions in the first model (*β* = −0.28 and *β* = 0.21, *p* = 0.001, respectively). The second model showed a significant improvement (Δ*F*(7,727) = 162.35, *p* < 0.000, Δ*R*^2^ = 0.226) and indicated a negative correlation between nurses' perceptions of emotional intelligence and occupational stress (*β* = −0.45, *t* = −12.8, *p* < 0.000). The nurses' sociodemographic characteristics in the first model explained 2.4% of the variance. The second model represented 25.0% of the variance when nurses' emotional intelligence perception was included.

**Conclusion:** Our study shows a novel framework that indicates a positive perceived effect of emotional intelligence on nurses' perceptions of occupational stress in Saudi Arabia. Our findings propose that emotional intelligence is a significantly effective mechanism against occupational stress.

## 1. Introduction

Globally, occupational stress (OS) is a formidable challenge in the fast-paced and high-stakes environment of healthcare settings. OS is highly prevalent among employees and varies according to their role and nature of their work. Various terms are used interchangeably to indicate stress and pressure in the workplace, such as *occupational stress*, *job-induced stress*, and *work-related* stress [1, 2]. The term “*occupational stress*” is used by psychologists and organizations to formally describe workplace stress and pressure [2]; hence, it has been adopted in the present study. For example, the OS was defined by World Health Organization [3] as “*the response people may have when faced with work demands and pressures that are not compatible with their knowledge and abilities*, *thus challenging their ability to cope*.” Unsurprisingly, it has been universally acknowledged that nurses work in particularly complex and highly stressful environments [4, 5]. Scholars have described the nursing profession as inherently stressful and anxiety-provoking, with recent reports highlighting its contribution to emotional exhaustion [6].

Considering the nursing perspective, OS has emerged as a significant challenge faced by nurses due to discrepancies between different expectations and realities, such as patient needs, coworker relationships, management demands, scope of practice, and resource availability [2]. A critical review by Chesak et al. [7] revealed that OS compromised the healthcare quality and jeopardized patients' safety. Furthermore, OS may not only affect patient well-being but also contribute to the economic collapse of healthcare organizations through massive attrition of resources, frequent absenteeism, poor performance, and staff resignation [8]. It is estimated that OS imposes a global financial burden ranging from $221.13 million to $187 billion annually [9, 10]. This substantial economic impact indicates increased healthcare costs, lost productivity, and expenses associated with recruiting and training new staff owing to high turnover rates [11]. Considering the exceptionally high stakes involved in healthcare settings, there is an urgent need for effective stress management strategies in the work environment. Therefore, researchers have recommended that nurses should establish protective measures against OS to ensure effective work performance [12].

Emotional intelligence (EI) was introduced by psychologists in 1990, Salovey and Mayer [13]. Mayer et al. [14] defined EI as “*the ability to carry out accurate reasoning focused on emotions and the ability to use emotions and emotional knowledge to enhance thought*” (p. 527). Over the last 30 years, several models have been developed and further revised to conceptualize the concept of EI. These models were classified into three distinct categories: ability-based models, trait-based models, and mixed-based models [15, 16]. In the ability-based EI models, the core tenet of EI emphasizes certain cognitive and emotion-related aptitudes [13, 14]. While in the trait models, EI is considered as a blend of personality dispositions and emotion-related capabilities [17].

In this research, we adopted Goleman's EI model [18]. This mixed-based model recognizes EI as a synthesis of social and emotional qualities, rather than merely a sole concept [18]. Goleman et al. [18] described EI as an adaptive competence that underpins optimum workplace performance. Although EI model of Goleman proposed a less scientific and more abstract conceptualization of EI [18], it was primarily espoused in empirical research [18–20]. The adaptive function of EI competence is a pillar in the management of OS [19, 21]. In this context, nurses with higher EI can more effectively recognize, understand, and control their own emotions, as well as empathize with and manage the emotions of others, indicating that EI acts as an effective coping mechanism to ameliorate OS in daily work [22]. Therefore, it can be inferred that emotionally intelligent nurses possess significant resilience against stress. Considering this correlation, the idea of incorporating EI competence as a critical prerequisite in the nursing recruitment process is being increasingly encouraged [23].

Empirically, OS is a widespread phenomenon affecting nurses working in both developing and developed countries [23]. This implies that OS is a 21st-century health epidemic which increases nurses' vulnerability to various physical and psychological illnesses [4]. In the United States, Mazzella–Ebstein et al. [24] assessed the OS perception among newly hired oncology nurses. The findings indicated that all recruited nurses experienced moderate to severe OS levels [4]. Despite using various measures to assess OS among nurses, the situation in developing countries is comparable. For instance, Chaudhari et al. [25] indicated that 88.6% of the recruited Indian nurses were severely stressful due to work conditions. Similar results of 89% and 81% were reported from Pakistan [26] and Iraq [27], respectively.

Unfortunately, OS has been increasing and has become an alarming threat to the Saudi healthcare system due to several factors, including patient-care demands and the absence of a clear scope of nursing practice [23, 28]. It has been widely reported among nurses in various wards and departments within healthcare institutions, indicating its widespread prevalence in the healthcare sector. For example, Almazan et al. [29] reported an extremely high perception of OS among acute care nurses. Alharbi and Alshehry [28] found that the majority of Saudi nurses in intensive care unit (ICU) experienced moderate to high levels of OS perception. Furthermore, OS has been reported among nurses in psychiatric units [30], palliative care units [31], and primary care centers [32].

Despite considerable evidence from Western studies that confirms the positive role of EI in mitigating the OS perception among nurses [3, 5, 9], existing knowledge on this phenomenon is still quite fragmented and scarce in Saudi Arabia. These inconsistencies in understanding the role of EI in managing OS among nurses in Saudi Arabia could lead to a practical gap that negatively affects patient care and safety. Without a clear integration of EI strategies into the nursing workforce, nurses may be less equipped to effectively manage OS, which could impact their decisions, clinical reasoning, and overall ability to provide safe and effective care. Addressing this gap is crucial for improving nurses' resilience and well-being. The findings of the present study could be instrumental in designing stress management programs or tailored policies to improve nurses' perceptions. The present study addressed the following questions to guide the research focus and establish a clear investigative framework.

  Q1: What are the perceived levels of EI and OS among a sample of nurses in Saudi Arabia?
  Q2: To what extent do the perceived levels of EI and OS vary between nurses when grouped according to control variables?
  Q3: How does the correlation between nurses' perceptions of EI and OS manifest when the effects of control variables are isolated?
  Q4: How much variance in nurses' OS perception can be accounted for by their scores on EI?

## 2. Methods

### 2.1. Study Design

A predictive correlational design was used since it is suitable and valid for determining the presence of predictive relationships between nurses' sociodemographic characteristics, their self-perceived level of EI, and their self-perceived level of OS. The study was conducted between August 25, 2022, and September 30, 2022.

### 2.2. Sample/Participants

A convenience sampling method was used to recruit licensed nurses registered with the Saudi Commission for Health Specialties, who had been employed as bedside staff nurses for more than 2 years. In contrast, managerial nurses, supervisors, clinical instructors, dependent and student nurses, and other nursing professionals performing nonclinical duties were excluded due to their significantly different scope of practice and concerns compared to bedside staff nurses. Additionally, staff nurses who were legally suspended or refused to participate in this study were excluded because their circumstances compromised their eligibility to adequately represent the target population.

Power analysis using G ∗ Power 3.1 TM software ™ was performed to determine the sample size required to produce statistically significant results. Considering a Cohen's medium effect size (*f*^2^ = 0.15), a power level of 95%, and a significance level of 0.05 as input parameters for a multivariate multiple regression analysis with seven predictors, the analysis indicated that a sample size of 153 nurses would be adequate to infer statistical significance, should it exist.

### 2.3. Measures

Data was collected using two self-administered instruments along with an appropriate sociodemographic questionnaire to meet the study objectives. A sociodemographic questionnaire was used to collect information on several control variables, including age, sex, tenure, area of work, level of education, and nationality. The included work areas were medical/surgical (Med/Surgical), Obstetrics Gynecology (OB/GYN), and Critical Care. Furthermore, all nurses working in the ICU and the High Dependency Unit were grouped under the category “critical care,” given the similarities in their scope of practice and clinical policies.

The first questionnaire was the Schutte Self-Report Emotional Intelligence Test (SSEIT), which was established to assess the self-perceived level of EI among nurses through 33 items [33]. The SSEIT measures the exclusive merging of noncognitive emotional talents, personal dispositions, and competences. Operationally, the items on the SSEIT are assessed on a 5-point scale that ranges from “*strongly disagree*” to “*strongly agree*.” It includes three items with reverse scoring (items 5, 28, and 33). The overall SSEIT scores vary between 33 and 165; scores < 110 represent poor EI, scores between 111 and 137 indicate average EI, and scores >138 indicate a high level of EI perception [34]. Several studies have determined the reliability of SSEIT with Cronbach's alpha values ranging from 0.88 to 0.93 [22, 23]. In our study, the internal consistency of SSEIT was found to be 0.91%. Permission was obtained to use the SSEIT in the present study.

Nursing Stress Scale (NSS) was also utilized to assess self-perceived OS among nurses [35]. The NSS consists of 34 items that are assessed on a 5-point scale and ranges from “never” to “very frequently.” Each item on the scale is assigned a numerical score from 0 to 3, yielding a total possible score ranging from 0 to 102. It is reported that an NSS total score of 39 indicates low stress perception, a score ranging from 40 to 62 signifies moderate stress, and a score greater than 63 stands for severe occupational stressful situation [36]. NSS was found to be reliable in early studies with *α* coefficient ranging from 0.81 to 0.94 [23, 36]. In the present study, its internal consistency assessed using *α* coefficient was 0.89. Permission was obtained to use NSS in the present study.

### 2.4. Data Collection

This study was conducted at two major hospitals that form the Taif Health Cluster in Saudi Arabia, King Abdulaziz Specialist Hospital (KAASH) and King Faisal Medical Complex (KFMC). Each hospital has a capacity of 600 beds. The KAASH and KFMC feature a unique culturally diverse population of nurses. The data collection process began after obtaining the relevant administrative approvals for the study proposal. The data collection process at KAASH and KFMC began simultaneously in August 2022 and continued through October 2022.

With the cooperation of three nurse volunteers, 500 self-administered questionnaires were distributed to nurses in five active working areas in each hospital. Paper brochures and gentle reminder emails were used to increase response rates. The head nurses sent reminder emails to encourage them to respond. Additionally, brochures and emails instructed respondents to return their written informed consent and responses in secured boxes at the nursing stations in each ward.

### 2.5. Ethical Considerations

Before beginning the data collection process, the principal investigator ascertained all required approvals. The Institutional Review Board of the Saudi Ministry of Health was approved this study (reference: HAP–02–T–067). Furthermore, permission to collect data from KAASH and KFMC was obtained from the respective nursing offices. In accordance with the Saudi National Committee Bioethics Guidelines and the ethical norms of the Declaration of Helsinki, we ensured that participation in this study was entirely voluntary and that all participants could withdraw at any point, for any reason, without any consequences. Therefore, the nature of the current study, its importance, the participation process, sample selection process, participants' rights, risks, benefits, and data confidentiality were explained to the candidates through a paper covering each questionnaire. Additionally, all participants provided their written informed consent before participating. Data anonymity and confidentiality were ensured by securing hard copies in a password-protected cabinet and storing the digital data on a password-protected computer.

### 2.6. Statistical Analysis

IBM® SPSS® Statistics for Windows v.25 [37] was used to input, process, and analyze data. In case of missing data, the data were treated according to established rules of thumb [38, 39]. This may involve the use of imputation techniques or a complete case analysis (CCA). Descriptive statistics were conducted to define the sample characteristics and describe the perceived amounts of EI and OS. Inferential statistics were used to reach statistical conclusions at a significance level (*α*) of 5%. Specifically, an independent *t*-test was used to assess the associations between gender and educational level with perceived levels of EI and OS. Meanwhile, a one-way ANOVA test was conducted to assess the associations between age groups, tenure, area of work, and nationalities with perceived levels of EI and OS. A linear transformation approach is suggested for highly skewed data [40].

To draw rigorous statistical conclusions, we ascertained that the regression assumptions were fulfilled. To confirm multivariate normality, we plotted the multivariate residuals on a scatterplot. The predicting values versus residuals on a scatterplot (*zpred* vs. *zresid*) were used to assess homoscedasticity and linearity. The Durbin–Watson test was performed to determine the autonomy of the error. Additionally, we utilized the variance inflation factor (VIF) equation to confirm the absence of multicollinearity as follows [38]:

(1) $$\begin{array}{c} \text{VIF}=\frac{1}{1-R2} \end{array}$$

Multivariate analysis was performed using a hierarchical linear regression with two models to adjust for the effects of nurses' control variables and determine the predictors of OS perception. In Model 1, we regressed age, sex, tenure, educational level, and nationality against perceived OS. In Model 2, nurses' perceptions of EI were regressed against their perceived OS while maintaining the control variables as constant. The generalizability of the final model was evaluated using the *R*-squared (*R*^2^) and adjusted *R*-squared (^adj^*R*^2^) values [41]. Additionally, we established the following predictive mathematical equation [41]:

(2) $$\begin{array}{c} {y=a+b}_{1}x_{1}{+b}_{2}x_{2}+℮, \end{array}$$

where *y* represents the predicted value of the dependent variable, *a* is the constant, and *b*_1_ signifies the estimated regression value for the independent variable (*x*_1_).

## 3. Results

From the cohort of 1321 registered nurses employed on a full-time basis at KAASH and KFMC, 1000 received invitations to participate in this study. Approximately 758 surveys were collected, with a response rate of 75.8%. Among the collected surveys, 24 were incomplete, leaving 734 valid cases for analysis. Because invalid cases accounted for 3.2% of the total sample (less than 5%), CCA was used to analyze 734 cases and exclude the 24 invalid cases.

Table 1 describes the biographical features of our sample. Of the sample, 67.6% constituted by females. Nurses aged 31–40 years constituted more than half of the sample (60.6%). Almost half of the participants (43.5%) had a tenure of 11–20 years; the sample included 116 senior nurses. One-third of the participants were Med/Surgical nurses (*n* = 226), whereas the least populated group was recruited from the OB/GYN ward. A majority of the sample (61.6%) had earned a bachelor of science in nursing. We recruited nurses of various nationalities: Filipino (35.2%), Indian/Pakistani (27.9%), Saudi Arabian (26.7%), and other nationalities (10.2%).

Table 2 indicates that the nurses in Taif had an adequate level of EI and a moderate perceived amount of OS with mean scores of 147.66 (17.2) and 40.5 (19.7), respectively. A significant difference in nurses' EI perceptions based on age was identified through one-way ANOVA; *F*(2,734) = 37.5, *p* < 0.01. The *Scheffé* post hoc test indicated that nurses' EI perceptions vary significantly with age. In contrast, although age affected EI perceptions, it did not have a similar effect on nurses' OS perception.

Furthermore, Table 2 shows significant differences in nurses' EI perception when grouped by their area of work; *F* (3,733) = 5.48, *p* < 0.001. Notably, Med/Surgical nurses exhibited the highest mean EI score (150.75 ± 15.9), while ICU nurses showed the lowest (143.88 ± 18.7). The *Scheffé* test confirmed that ICU nurses' perception of EI was significantly lower than the perception of EI among nurses in other areas (*p* < 0.001). ICU nurses, who reported the lowest EI perception, experienced more OS (52.10 ± 14.1). On the contrary, Med/Surgical nurses, who had the highest EI, reported the lowest OS perception (34.75 ± 15.3). The *Scheffé* test also confirmed that the OS perceived by ICU nurses was significantly higher than that reported by nurses in other areas (*p* < 0.000). Although women comprised two-thirds of the participants, no significant sex-related differences were observed in nurses' perceptions of EI and OS. Furthermore, inferential statistics revealed that tenure, educational level, and nationality did not significantly influence nurses' perceptions of EI or OS.

**Figure 1:**
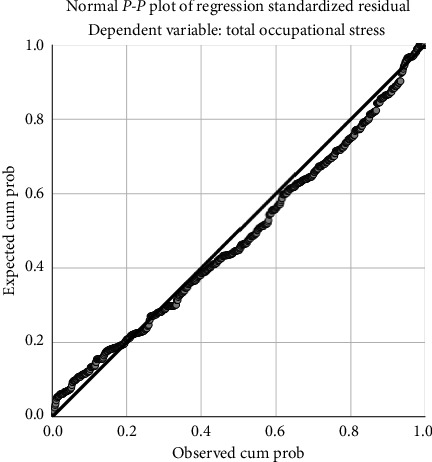
Normal probability plot (*P*–*P*) of the regression standardized residuals.

No contraventions were reported based on the findings of the tests used for establishing the regression assumptions. Our dataset achieved multivariate normality as the residuals clustered closely around the line of normality (Figure 1) [38].

**Figure 2:**
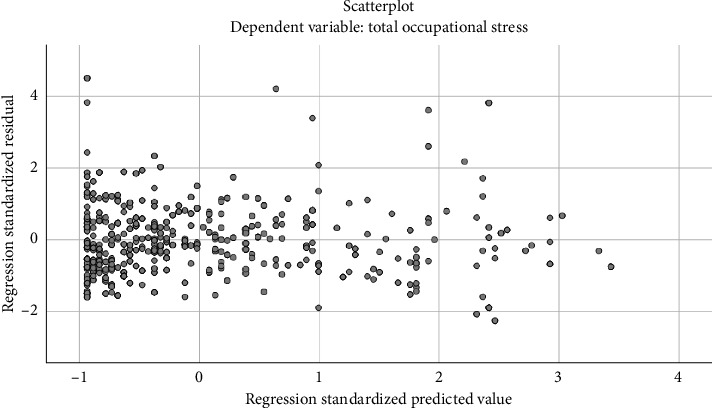
Scatterplot representing predicted values against errors.

Furthermore, the homoscedasticity of our data was confirmed since Figure 2 shows the scattered array of dots around the horizontal line [40]. The Durbin–Watson test [38] verified the independence of the residuals in all models, with values of 1.87 and 1.93. Furthermore, the absence of multicollinearity was assumed given that the VIF values ranged from 1.0 to 1.17 [40].

In Table 3, a hierarchical regression technique with two models was used to assess the predictive relationship between nurses' perceptions of EI and OS, while controlling for the effects of their sociodemographic variables. Table 3 shows that nurses' age and area of work significantly predicted their OS perception (*β* = −0.28, *t* = −1.88, *p* = 0.006 and *β* = 0.21, *t* = 3.25, *p* = 0.001, respectively). When these predictors were controlled, Model 2 significantly indicated a negative correlation between nurses' perception of EI and OS (*β* = −0.45, *p* < 0.000); OS perception significantly decreases as EI increases and vice versa.

Table 4 indicates that Model 1 was significant, *F*(6,728) = 2.95, *p* = 0.007, *R*^2^ = 0.024. Model 2, which included nurses' perceptions of EI, was also significant, *F*(7,727) = 165.3, *p* < 0.000, *R*^2^ = 0.25. In Table 4, a significant improvement from the first model was indicated, Δ*F*(1,727) = 162.35, *p* < 0.001, Δ *R*^2^ = 0.226. In general, nurses' age and working area in healthcare settings in the first model explained 2.4% of the variance. The regression equation for the first model is as follows:

(3) $$\begin{array}{c} \text{OS}=48.43-2.41\;\left(\text{age}\right)+2.17\;\left(\text{work area}\right) \end{array}$$

On the other hand, when nurses' perception of EI was included in the second model, it represented 25.0% of the overall OS variance. Hence, the equation for the second model was developed as follows:

(4) $$\begin{array}{c} \text{OS}=106.5-0.44\;\left(\text{EI perception}\right) \end{array}$$

The cross-validation of the final model confirmed that the difference between the values of *R*^2^ and ^adj^*R*^2^ was very small (0.250–0.241 = 0.004), indicating that if the final model were drawn from the population, the variance in the outcome would decrease by 4%.

## 4. Discussion

The global interest in EI originated from the prevailing belief that successful individuals possess unique competencies beyond mere intellectual ability, which significantly enhance their success in life. This is justified by the established role of EI in accurately perceiving, managing, and utilizing emotions, thereby improving one's quality of life. This study examined the perceived levels of EI and OS among nurses in Saudi Arabia. It also explored the extent to which EI can serve as a predictive factor for OS to determine whether higher levels of EI correlate with reduced levels of stress in the nursing profession.

In terms of EI perception, our findings revealed that a vast proportion of participants rated themselves as emotionally intelligent. These findings are congruent with those of earlier studies conducted in Saudi Arabia [42, 43]. These studies endorse EI competency as the fundamental essence of the nursing profession in Saudi Arabia [23, 42, 43]. Therefore, we argue that achieving excellence in nursing practice necessitates that nurses use EI to build successful relationships, utilize effective communication, exercise self-restraint, empathize with others, and remain mindful of their own emotions and those of others, particularly in emotionally exhausting situations. In contrast, studies from Iran [44] and India [45] found lower perceptions of EI among nurses. These variations in the perception of EI could be due to various background factors such as differences in cultural, educational, and healthcare systems.

On the other hand, concerning the perception of OS, our findings showed that nurses perceived OS at moderate levels. Previous studies from both developed and developing countries have reported similar results [5, 22, 43]. These studies indicated that nurses experience moderate to high levels of OS. Given the inherent challenges of the nursing profession, it is not surprising that it is often associated with stress and anxiety [4]. While there is an increasing number of studies emerging on the causes of OS among nurses, the reported OS predictors are limited to involvement in work duty rotation [46], massive workload [47], death and dying situations [27, 35, 36], lack of resources [26, 35], insecure workplace environment [32], and lack of communication [26, 30].

The roles of personal traits and professional characteristics in developing EI competence are equally important. In relation to EI, nurses' age, gender, education, income, and marital status were the common investigated demographic characteristics, while years of clinical experience, shift type, working area, and job position were the primary professional characteristics examined [23, 24, 34]. Our results showed that most senior nurses perceived themselves as the most emotionally intelligent, followed by those in their mid-career stages, while the youngest nurses reported the lowest amount of EI. These findings are analogous to those reported in previous studies, all of which indicate that the nature of the association between EI and nurses' age is no longer a matter of conjecture, as EI is a maturation process that develops with age [5, 23, 46]. Notably, the contradictory studies cannot be dismissed [48–50]. Aldossary et al. [48] and Ezenwaji et al. [50] concluded that EI can be acquired and developed at any age, indicating that it is not specific to a particular age group. However, these studies presented contrasting results that could stem from unrepresentative and biased samples.

Although multiple previous studies concur on the proposition that junior nurses are the most vulnerable to OS [51], we did not observe a significant effect of nurses' age on perceptions of OS. These findings are consistent with those of other studies that challenge the notion that young and newly hired nurses are more prone to OS [5, 50]. Contradictory studies argue that it is reasonable to suggest that OS levels might gradually diminish as age and maturity increase [51, 52]. From our side, we propose that our results can be attributed to the proper implementation of General Nursing Orientation programs for newly hired nurses in the context of our study.

Given that nurses operate in various medical wards or units, each with different work demands in healthcare institutions, we evaluated the influence of this contextual factor on nurses' perceptions of both EI and OS. Our findings indicated that Med/Surgical nurses perceived the highest level of EI, whereas ICU nurses perceived the lowest. In contrast, ICU nurses who exhibited the lowest perceived EI were occupationally stressed, while Med/Surgical Nurses with higher EI had the lowest OS. These results are consistent with those reported in Saudi [24] Iranian studies [53, 54]. These studies have empirically affirmed that the nature of work is fundamental in developing the perception of OS among nurses [24, 54]. These studies concluded that the patients' nature and demands might predispose nurses to critical amount of OS [24, 31, 32, 53].

Despite the common belief that females are considerably more emotionally intelligent than male nurses [55], our findings indicate that EI is not sex specific. These results align with findings from studies with unbiased and representative gender samples [44, 56]. Notably, some of the contradictory studies predominantly composed of female participants indicate a clear criticism to their external validity [22, 50]. Similarly, sex did not significantly affect nurses' perceptions of OS. These findings are consistent with previous research conducted in Saudi Arabia [23], Iran [53, 54], and China [57].

Similarly, tenure in clinical experience did not affect nurses' perceptions of EI or OS. These outcomes are congruent with those of Aldossary et al. [48] and Ezenwaji et al. [50], who found that the influence of clinical experience on nurses' perceptions of EI and OS was negligible. Additionally, we did not observe marked variations in nurses' perceptions of EI and OS when participants were classified according to their educational level and nationality. These findings are analogous to those of earlier Saudi [58], Iranian [59], and Chinese studies [57].

Importantly, our study found a negative association between nurses' perceptions of EI and OS. In other words, we established that more emotionally intelligent nurses reported the lowest perception level of OS. In the context of Goleman's EI model [18], it can be argued that emotionally intelligent nurses are better equipped to understand both their own emotions and those of others, build successful therapeutic nurse–patient relationships, and empower their team members, particularly in emotionally challenging situations, which in turn helps them cope more effectively with OS. Empirically, our results established that sufficient EI can act as an effective behavioral buffer and coping mechanism against OS [23, 31, 43]. Similar associations have been identified in previous studies conducted in Iraq [60], Iran [53], India [54], and the United States [22]. These studies lend evidence to the argument that EI is an inherent competence distinguishing nurses as effective leaders, innovators, and competent managers findings lend evidence to support the argument of EI is an inherent competence distinguishing nurses as effective leaders, innovators, and competent managers [19].

This study highlights crucial implications for nursing administration and education, emphasizing the need for adaptive leadership and curriculum reform to address emerging OS. For nursing administration, this study suggests a strategic assessment of workforce management and organizational culture to create an emotionally intelligent environment. Given the critical role of EI in mitigating nurses' OS, it is necessary to embed EI principles in leadership practices and workplace policies. This approach aims to develop a supportive and resilient nursing workforce equipped to manage the complexities of healthcare delivery while ensuring a healthier and more productive workplace. Additionally, Saudi Board for Accreditation of Healthcare Institutions is encouraged to incorporate EI competence into the evaluation standards to improve the safety and quality of care. In accordance with Goleman's EI model [18], it is evident that EI competence is not merely an innate talent, but rather a skill that can be taught and learned. Hence, our findings could assist nurse educators and curriculum developers in integrating EI into nursing curriculum to equip future nurses with resilience skills.

Our study, while insightful for nursing management, practice, and education, faces limitations such as its lack of generalizability due to a convenience sample from a single city, reliance on self-reported data prone to bias, and focus on a mixed EI model only. Future research should seek broader demographics, employ objective measures or qualitative approaches, explore various EI models, investigate EI subdimensions, and consider social intelligence. Additionally, addressing the scarcity of EI studies in nursing education and identifying additional influential factors can provide a more comprehensive understanding and effective interventions for OS.

## 5. Conclusion

The nursing profession is often characterized by high levels of stress, anxiety, and emotional exhaustion due to its demanding nature. OS has been recognized as a major occupational health problem in the 21st century, affecting workers in various sectors, including nursing. Beyond its effects on the organizational economy, it places patient safety at risk.

The present study found that nurses' age, working area, and perceived EI were significant predictors of their OS perceptions. Additionally, it indicated a negative association between nurses' perceptions of EI and OS, suggesting that more emotionally intelligent nurses experienced less OS due to their work conditions and vice versa.

This study presented a novel model for mitigating OS among nurses in the workplace. This model may help to develop relevant policies and protocols to improve the quality and safety of healthcare services. From an educational perspective, such competence is not a mere innate talent but can be acquired through schooling and education.

## Figures and Tables

**Table 1:** Sociodemographic profile of our participants.

Variable	Participants (*n* = 734)		
	Categorize	*F*	%
Gender	Male	238	32.4
	Female	496	67.6

Age	25–30 years	94	12.8
	31–40 years	445	60.6
	41–50 years	195	26.6

Tenure	2–10 years	299	40.7
	11–20 years	319	43.5
	21–30 years	116	15.8

Working area	Medical/surgical	226	30.8
	OB/GYNEa	158	21.5
	ICU	180	24.5
	Other wards	170	23.2

Educational level	Diploma in nursing	282	38.4
	Bachelor in nursing	452	61.6

Nationality	Saudi	196	26.7
	Filipino	258	35.2
	Indian/Pakistani	205	27.9
	Other	75	10.2

^a^Obstetrics and gynecology.

**Table 2:** Results of descriptive and inferential statistics.

	Categories	*N*	EI1 *X* ± SD	OS2 *X* ± SD
Age groups	25–30 years^a^	94	136.4 ± 19.9	44.6 ± 17.3
	31–40 years^b^	445	146.8 ± 16.4	39.8 ± 18.2
	41–50 years^c^	195	154.9 ± 13.7	40.0 ± 23.5
	Sig.		*F* = 37.5^∗^	*F* = 3.59
	Scheffeé		c > b > a	

Gender	Male	238	147.92 ± 17.6	41.84 ± 20.7
	Female	496	147.54 ± 16.9	39.92 ± 19.2
	Sig.		*t* = 0.26	*t = 0*.*233*

Tenure	2‒10 years	299	147.39 ± 17.3	40.54 ± 21.2
	11‒20 years	319	147.12 ± 17.2	41.48 ± 18.1
	21‒30 years	116	149.86 ± 16.9	37.96 ± 20.1
	Sig.		*F = 1*.*15*	*F* = 1.36

Working area	Medical/surgicald	226	150.75 ± 15.9	34.75 ± 15.3
	OB/GYNEe	158	146.71 ± 16.9	40.63 ± 16.5
	ICUf	180	143.88 ± 18.7	52.10 ± 14.1
	Otherg	170	149.02 ± 16.4	35.92 ± 17.2
	Sig.		*F = 5*.*48*^∗∗^	*F* = 34.43^∗∗^
	Scheffeé		f < d, e, g	f > d, e, g

Edu. Level	Diploma	282	147.93 ± 17.5	41.40 ± 20.4
	Bachelor	452	147.50 ± 16.9	40.01 ± 19.2
	Sig.		*t = 0*.*336*	*t* = 0.933

Nationality	Saudi	196	147.75 ± 18.4	41.59 ± 19.6
	Filipino	258	147.41 ± 17.2	41.47 ± 22.167
	Indian/Pakistani	205	146.82 ± 17.1	39.01 ± 16.9
	Other	75	150.61 ± 13.2	38.80 ± 18.0
	Sig.		*F = 0*.*925*	*F* = 0.985

Total		734	147.66 ± 17.2	40.5 ± 19.7

^1^Emotional intelligence.

^2^Occupational stress.

^a^Young adults.

^b^Established adults.

^c^Mature adults.

^d^Medical and surgical departments.

^e^Obstetrics and gynecology department.

^f^Intensive care units.

^g^Other nursing areas.

^∗^ *p* < 0.01.

^∗∗^ *p* < 0.001 (2-tailed).

**Table 3:** Regression analysis results^a^.

Model	Predictors	*b*	Std. error	*β*	*T*	*Sig*.	CI 95% [lower, upper]
1^b^	(Constant)	48.43	4.74		10.2	0.000	[39.1, 57.7]
	Age	–2.41	1.27	–0.28	–1.88	0.006	[–4.92, 0.10]
	Gender	–1.66	1.55	–0.03	–1.07	0.29	[–4.70, 1.39]
	Tenure	–0.38	1.01	–0.016	–.38	0.70	[–2.37, 1.60]
	Working area	2.17	0.66	0.21	3.25	0.001	[0.86, 3.47]
	Nationality	–0.89	0.76	−0.04	–1.17	0.24	[–2.39, 0.61]
	Academic level	–1.55	1.49	–0.04	–1.04	0.29	[–4.47, 1.37]

2^c^	(Constant)	106.5	6.23		17.11	0.000	[94.3, 118.8]
	Total EI	**–0.44**	**0.04**	**–0.45**	**–12.8**	**0.000**	[–0.52, −0.38]

*Note:* Bold values indicate significance.

^a^ *n* = 734.

^b^Durbin–Watson = 1.86.

^c^Durbin–Watson = 1.93.

**Table 4:** Model fit measures.

Model	Occupational stress^a^						
	*R* ^2^	*ΔR* ^2^	a ^dj^ *R* ^2^	*F*	*df* 1	*df* 2	*Sig*
Model 1^b^	0.024		0.019	2.95	6	728	**0.007**
Model 2^c^	0.250	**0.226**	**0.210**	165.3	7	727	**0.000**

*Note:* Bold values indicate significance.

^a^ *n* = 734.

^b^Predictors: (constant), age, gender, tenure, nationality, working area, educational level.

^c^Predictors: (constant), age, gender, tenure, nationality, working area, educational level, EI.

## Data Availability

The data that support the findings of this study are available from the corresponding author upon reasonable request.
